# A Physician’s Lifetime

**Published:** 1881-05

**Authors:** 


### A PHYSICIAN'S LIFE TIME.

If a young graduate on the day of first opening an office will school himself to look wise and say nothing, have a cast of brass made for his face, encase his hide, heart, and conscience with the skin of a rhinoceros, he will infallibly get practice, grow rich, and live a long time. But if he begins his professional career with a determination to do all he possibly can to save the life of the one intrusted to him, even at the peril of his own, to abhor all pretence and trickery, and to act with candid conscientiousness towards those who repose confidence in him, the result will be poverty and a premature death, in a very large number of cases; and this is the reason why so many physicians of education and talent either fail to live by their profession or die before their time, in the vain struggle for that respectable style of living which belongs to their calling. No class of men, the clergy not excepted, give as much pecuniary aid in proportion to their means, to suffering humanity, as a physician engaged in the general practice of medicine, and no class of men are as often and as grossly imposed upon. Dr. Mott, the Nestor of our profession, once remarked, and with great truth, to a graduating class: "*Young gentlemen, have two pockets made, a large one to hold the insults, and a small one for the fees.*"

According to statistical returns, out of every hundred persons, engaged in different callings, living beyond seventy years, there were, in each calling,

42 Clergymen,
40 Farmers,
35 Merchants,
35 Office holders,
32 Soldiers,
29 Lawyers,
27 Artists,
27 Teachers,
24 Physicians.

That is to say, clergymen have nearly two chances to one for a long life over the physician.

